# ST-Elevation Myocardial Infarction Despite Adequate Anticoagulation in a Patient With Triple-Positive Antiphospholipid Syndrome: A Therapeutic Dilemma in a Resource-Limited Setting

**DOI:** 10.7759/cureus.87433

**Published:** 2025-07-07

**Authors:** Kajananan Sivagurunathan, Nishadi Perera, Anuranga Senanayake, Jeyakanth Thangarajah

**Affiliations:** 1 Internal Medicine, District General Hospital, Kilinochchi, LKA; 2 Cardiology, District General Hospital, Kilinochchi, LKA

**Keywords:** acute st myocardial infarction, anti-phospholipid antibodies, anti-phospholipid antibody syndrome (aps), percutaneous coronary intervention, primary pci, st-elevation myocardial infarction (stemi)

## Abstract

Antiphospholipid syndrome is a prothrombotic autoimmune disorder that can lead to arterial thrombotic events such as acute myocardial infarction. We report a case of a 40-year-old female with triple-positive antiphospholipid syndrome on therapeutic warfarin who presented with anterior ST-elevation myocardial infarction. With an international normalized ratio of 2.3, thrombolysis was contraindicated, and primary percutaneous coronary intervention was unavailable due to resource limitations. She was managed conservatively with enoxaparin, dual antiplatelet therapy, and supportive care. Elective angiography revealed complete occlusion of the left anterior descending artery, and viability testing guided subsequent percutaneous coronary intervention, resulting in good functional recovery. An individualized antithrombotic regimen with a clear timeline was offered after a multidisciplinary team discussion. This case highlights the therapeutic challenges of ST-elevation myocardial infarction in patients with anticoagulated antiphospholipid syndrome, particularly in settings without primary percutaneous coronary intervention.

## Introduction

Antiphospholipid syndrome (APLS) is an autoimmune prothrombotic disorder characterized by the presence of antiphospholipid antibodies and clinical manifestations such as arterial or venous thrombosis and pregnancy morbidity [1]. While venous thromboembolism is more common, arterial events such as stroke and myocardial infarction can also occur, particularly in younger patients, often in the absence of traditional cardiovascular risk factors [2]. Studies suggest that approximately 2.8% to 5.5% of acute myocardial infarction cases in young people are linked to APLS [3]. Typically, acute myocardial infarction associated with APLS presents in patients during their thirties [3].

ST-elevation myocardial infarction (STEMI) in patients with triple-positive APLS poses a significant therapeutic challenge, especially when it occurs despite therapeutic anticoagulation with warfarin. In such cases, thrombolysis is often contraindicated due to bleeding risk, and access to primary percutaneous coronary intervention (PCI), the preferred reperfusion strategy, may be limited in resource-constrained settings [4].

This case highlights the real-world difficulties of managing acute coronary syndromes in anticoagulated APLS patients and underscores the importance of individualized, multidisciplinary decision-making in settings where standard care pathways may not be feasible.

## Case presentation

A 40-year-old female with a known diagnosis of triple-positive APLS presented to our facility with the acute onset of chest pain. Her past medical history was significant for multiple second-trimester miscarriages and two episodes of intrauterine fetal demise, fulfilling the obstetric criteria for APLS. Additionally, she had a prior unprovoked deep vein thrombosis of the right lower limb, for which she was on long-term oral anticoagulation with warfarin, with a target international normalized ratio (INR) of 2.0-3.0. She had no family history of atherosclerotic cardiovascular disease, no personal risk factors such as dyslipidemia, hypertension, or diabetes, and no history of oral contraceptive use.

**Figure 1:**
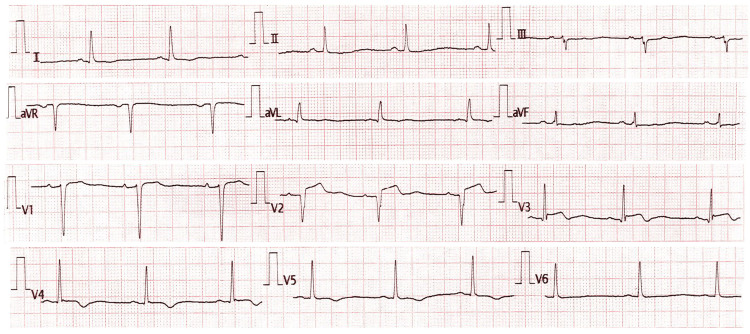
The 12-lead electrocardiogram revealed ST elevation in leads V1 to V4, along with Q waves in V1 and V2 and terminal T wave inversions in leads V3 to V5, indicative of an evolving anteroseptal ST-elevation myocardial infarction.

She reported a central, tightening-type chest pain lasting for five hours, associated with autonomic symptoms, including diaphoresis and nausea. On examination, her vital signs were stable: blood pressure, 130/84 mmHg; pulse rate, 88 beats per minute; respiratory rate within normal limits; and oxygen saturation, 98% on room air. Lung auscultation revealed clear lung fields. A 12-lead electrocardiogram demonstrated ST-segment elevation in leads V1 to V4, Q waves in V1-V2, and terminal T wave inversions in V3-V5, consistent with an evolving anteroseptal STEMI (Figure 1). High-sensitivity troponin I was 9432 ng/L (<19 ng/L), confirming myocardial injury. Her INR at the time of presentation was 2.3, rendering her ineligible for thrombolytic therapy due to the high risk of bleeding complications. Unfortunately, as with most centers in the region, our hospital lacks facilities for primary PCI, which would otherwise be the optimal treatment modality in this setting.

Considering these constraints, a multidisciplinary discussion was held with the cardiology team, and a decision was made to initiate conservative medical management. The patient was started on enoxaparin 60 mg subcutaneously twice daily, along with dual antiplatelet therapy consisting of aspirin 75 mg and clopidogrel 75 mg once daily. Additionally, atorvastatin 40 mg once daily was prescribed.

**Figure 2:**
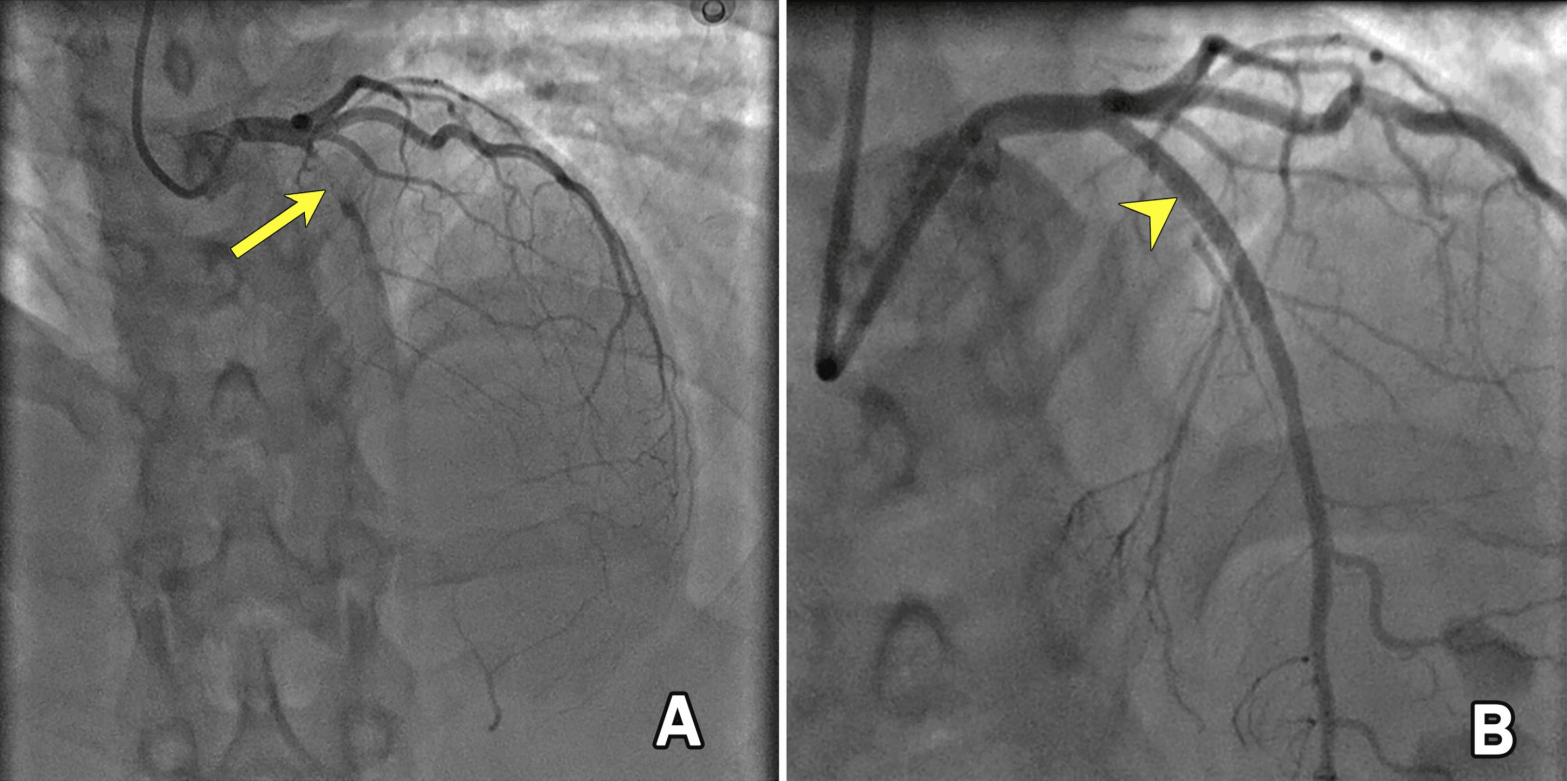
Initial elective coronary angiogram following myocardial infarction (A) shows complete occlusion of the left anterior descending artery (arrow), while the post-percutaneous coronary intervention angiogram (B) demonstrates successful revascularization (arrowhead) with restoration of flow in the left anterior descending artery.

A transthoracic echocardiogram revealed a left ventricular ejection fraction (LVEF) of 40%, with hypokinesia of the anterior wall segments. Warfarin therapy was temporarily discontinued, and bridging anticoagulation with therapeutic-dose enoxaparin was continued. It was decided to perform elective coronary angiography once the INR decreased to below 1.5. On day five of admission, with an INR <1.5, coronary angiography was performed. It revealed a complete occlusion of the left anterior descending artery (LAD) (Figure 2A), with no significant stenosis in the right coronary artery or left circumflex coronary artery. Given her stable condition and ongoing anticoagulation, the cardiology team opted to defer immediate revascularization and recommended viability assessment before intervention. Warfarin was resumed alongside clopidogrel, with a target INR range of 2.5-3.5, while aspirin was discontinued after one week due to a high risk of bleeding. Three weeks later, a dobutamine stress echocardiogram was performed, which demonstrated viable myocardium in the affected territory. Warfarin was subsequently withheld, and the patient was bridged again with enoxaparin in preparation for PCI. PCI was successfully performed, with restoration of flow in the previously occluded LAD (Figure 2B). A follow-up echocardiogram after the procedure showed significant improvement in left ventricular function, with an LVEF of 60%. We planned to continue warfarin with a target INR of 2.5 to 3.5 in combination with clopidogrel for one year. After one year, clopidogrel would be discontinued, and the target INR would be increased to 3.0 to 4.0. Her follow-up has been uneventful to date, with no recurrence of symptoms. The lipid profile revealed no significant abnormalities, and intravascular imaging, such as intravascular ultrasound or optical coherence tomography, was not performed due to its unavailability in our setting.

## Discussion

APLS is a prothrombotic autoimmune disorder characterized by the presence of antiphospholipid antibodies, including lupus anticoagulant, anticardiolipin, and anti-β2 glycoprotein I antibodies. Triple-positive APLS, defined by positivity for all three antibodies, confers the highest thrombotic risk and often warrants lifelong anticoagulation, typically with warfarin [5]. Despite therapeutic anticoagulation, thrombotic events may still occur in patients with APLS due to the aggressive nature of the disease. This is attributed to several factors, including persistent endothelial dysfunction, ongoing activation of the coagulation cascade, complement activation, and the presence of high-titer antiphospholipid antibodies that enhance platelet aggregation and interfere with natural anticoagulant pathways [5]. Additionally, triple-positive APLS is associated with a higher risk of recurrent thrombotic events, underscoring the limitations of standard anticoagulation and the need for individualized, intensified treatment strategies in select patients.

In this case, the patient presented with an anterior STEMI while on warfarin therapy with a therapeutic INR of 2.3. This posed a significant therapeutic dilemma. Thrombolysis, though recommended in the absence of primary PCI, was contraindicated due to the elevated INR and risk of major bleeding. Primary PCI, the preferred reperfusion strategy for STEMI, was not available in our region, reflecting a common challenge in resource-limited settings. Faced with these constraints, we opted for a conservative approach comprising dual antiplatelet therapy (aspirin and clopidogrel), therapeutic-dose enoxaparin, and close monitoring. Elective coronary angiography revealed complete occlusion of the LAD. Revascularization was appropriately deferred until functional assessment confirmed viable myocardium. In accordance with European Society of Cardiology (ESC) guidelines, a dobutamine stress echocardiogram was performed to assess myocardial viability before revascularization, in order to determine the presence of salvageable myocardium and guide the appropriateness of PCI in this patient [6]. Ultimately, PCI was successfully performed with restoration of flow and improvement in left ventricular function. In this patient, bridging with enoxaparin was used when warfarin was withheld to allow INR reduction before invasive procedures. This approach aimed to minimize thrombotic risk in a triple-positive APLS patient. While bridging strategies are commonly employed in such contexts, a detailed discussion of anticoagulation management protocols is beyond the scope of this case report. 

Management of acute coronary syndrome in APLS patients requires balancing ischemic and bleeding risks, especially when combining antiplatelets and anticoagulants. There is limited evidence guiding dual or triple therapy in such scenarios, and decisions must be individualized. In our patient, triple antithrombotic therapy with aspirin, clopidogrel, and warfarin was initiated. Aspirin was discontinued after one week due to bleeding concerns. Warfarin was continued alongside clopidogrel, with a target INR of 2.5-3.5. After one year, clopidogrel is planned to be discontinued, and the target INR is increased to 3.0-4.0 to optimize long-term thrombotic protection. These decisions were made following a multidisciplinary discussion and in accordance with the latest ESC guidelines. According to the ESC guidelines, in patients requiring long-term oral anticoagulation who are at high risk for ischemic events, triple antithrombotic therapy (dual antiplatelet therapy plus an oral anticoagulant) is recommended for one to four weeks. This should be followed by dual therapy with an oral anticoagulant and a single antiplatelet agent for up to 12 months, and subsequently oral anticoagulation alone beyond one year [7]. We planned to intensify the target INR from 2.5-3.5 to 3.0-4.0 after one year and discontinue clopidogrel based on an individualized, patient-tailored decision following multidisciplinary team discussion, although this approach is not supported by high-quality, evidence-based studies. Two randomized trials evaluating warfarin intensity in APLS patients with prior thrombosis found no benefit of high-intensity anticoagulation over standard-intensity therapy. A trial of 109 patients found that high-intensity warfarin (INR: 3.0-4.5) did not reduce thrombotic events compared to conventional therapy (INR: 2.0-3.0 or aspirin) but was linked to increased minor bleeding [8]. Similarly, another study with 114 patients showed higher recurrent thrombosis rates in the high-intensity group (INR: 3.1-4.0) than in the moderate-intensity group (INR: 2.0-3.0), with a similar bleeding risk [9]. Both studies support maintaining a standard INR target of 2.0-3.0 for long-term anticoagulation in APLS to balance efficacy and safety; however, high-intensity anticoagulation (INR: 3.0-4.0) was selected in our patient after multidisciplinary team discussion and individualized patient assessment.

Evidence suggests that direct oral anticoagulants are less effective than warfarin in preventing recurrent thrombotic events in patients with APLS, particularly among those with triple-positive serology or a history of arterial thrombosis [10]. Statins may be considered as adjunctive therapy in patients with APLS who experience recurrent thromboembolic events despite adequate anticoagulation, as emerging evidence suggests they may reduce proinflammatory and prothrombotic mediators [11,12]. In our patient, statin therapy was planned to be continued as part of the long-term management strategy. 

Hydroxychloroquine may be a valuable adjunctive therapy for patients with refractory cases. Hydroxychloroquine is routinely used in the treatment of systemic lupus erythematosus and has demonstrated potential in reducing thrombotic events in patients with coexisting systemic lupus erythematosus and APLS. However, in patients with primary APLS, as in our case, the supporting evidence remains limited [13,14]. Hydroxychloroquine was not offered to our patient.

## Conclusions

This case highlights the complex challenges in managing acute coronary syndrome in patients with triple-positive APLS, particularly in resource-limited settings without access to primary PCI. Despite therapeutic anticoagulation, thrombotic events can still occur, necessitating multidisciplinary coordination and a tailored approach to antithrombotic therapy in complex prothrombotic conditions such as APLS. Conservative medical management followed by elective PCI after viability assessment can be an effective strategy when immediate intervention is not feasible. This case also underscores the need for context-specific strategies in low-resource settings where standard interventions such as primary PCI are not readily available. Long-term management requires individualized anticoagulation strategies, guided by clinical judgment and current guidelines to optimize patient outcomes.
